# Supplementary material for: Synthesis and Antiproliferative Effect of Halogenated Coumarin Derivatives
Source: Molecules. 2022 Dec 14;27(24):8897. doi: 10.3390/molecules27248897 (PMC9786284; doi:10.3390/molecules27248897)

Supporting Information

Synthesis and antiproliferative effect of halogenated coumarin derivatives.

Tinuccia Dettori[1‡], Giuseppina Sanna[1‡], Andrea Cocco[2‡], Gabriele Serreli[1], Monica Deiana[1], Vanessa Palmas[1], Valentina Onnis[3], Luca Pilia[4], Nicola Melis[4], Davide Moi[2,3*], Paola Caria[1*] and Francesco Secci[2*].

[1]Department of Biomedical Sciences, University of Cagliari, Cittadella Universitaria, SS 554, km 4.5, 09042 Monserrato, Italy

[2]Department of Chemical and Geological Sciences, University of Cagliari, Cittadella Universitaria, SS 554, km 4.5, 09042 Monserrato, Italy

[3]Department of Life and Environmental Sciences, University of Cagliari, Cittadella Universitaria, SS 554, km 4.5, 09042 Monserrato, Italy

[4]Department of Mechanical, Chemical and Material Engineering, University of Cagliari. Piazza d'armi, 09123, Cagliari, Italy

[‡] Equally contributing authors.

[*]davide.moi2@gmail.com (D.M.); fsecci@unica.it (F.S.); paola.caria@unica.it (P.C.).

Table of contents

**1. General remarks**	**S2**
**2. Cyclic voltammetry diagram of coumarins 2k and 2h**	**S3**
**3. ^{1}H and ^{13}C NMR spectra of compounds 2a-2k**	**S4**

1. General remarks

Unless stated otherwise, respectively the synthesis of compounds 2a-k were performed at the indicated temperature in a round bottom flask equipped with a stirring bar and a condenser if needed. Commercially available reagents were used as received unless otherwise noted. The salicylaldehydes 1 used in this work were purchased from Sigma Aldrich, TCI of Fluorochem. ^{1}H NMR spectra were recorded on a Bruker Avance III HD 600 spectrometer (Bruker, Bremen, Germany) at 300.15 K using $CDCl_3$ (ref. 7.27 ppm), as a solvent. ^{13}C NMR were recorded on a Bruker Advance III HD 600 (Bruker, Bremen, Germany) at 126 MHz (ref. $CDCl_3$ 77.00 ppm) at 300.15 K using $CDCl_3$, as solvent. Chemical shifts (δ) are given in ppm. Coupling constant values (J) are reported in Hz. Infrared spectra were recorded on an FT-IR Bruker Equinox-55 spectrophotometer (Bruker, Bremen, Germany) and are reported in wavenumbers (cm^{-1}). Low Mass Spectra Analysis were recorded on an Agilent-HP GC-MS (E.I. 70eV). High Resolution Mass Spectra (HRMS) were obtained using a Bruker High Resolution Mass Spectrometer (Bruker, Bremen, Germany) in fast atom bombardment (FAB^+) ionization mode. Melting points were determined with a Büchi M-560 Analytical (Buchi. Flawil, Swiss). Thin layer chromatography was performed using 0.25 mm Aldrich silica gel 60-F plates. Flash chromatography was performed using Merk 70-200 mesh silica gel. Yields refer to chromatography and spectroscopically pure materials

2. Cyclic voltammetry of 2k and 2h

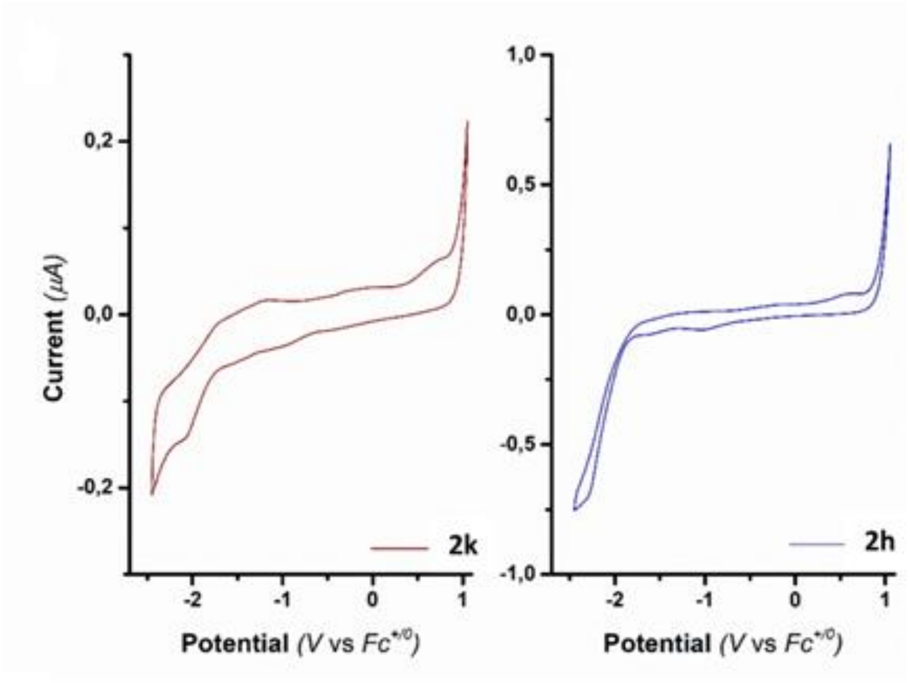

Figure S1. Cyclic voltammetry (CV) plots for coumarins 2k and 2h

3. ^{1}H and ^{13}C NMR characterization of compounds 2a-2k

2a: 6-fluoro-2-oxo-2*H*-chromene-3-carbonitrile

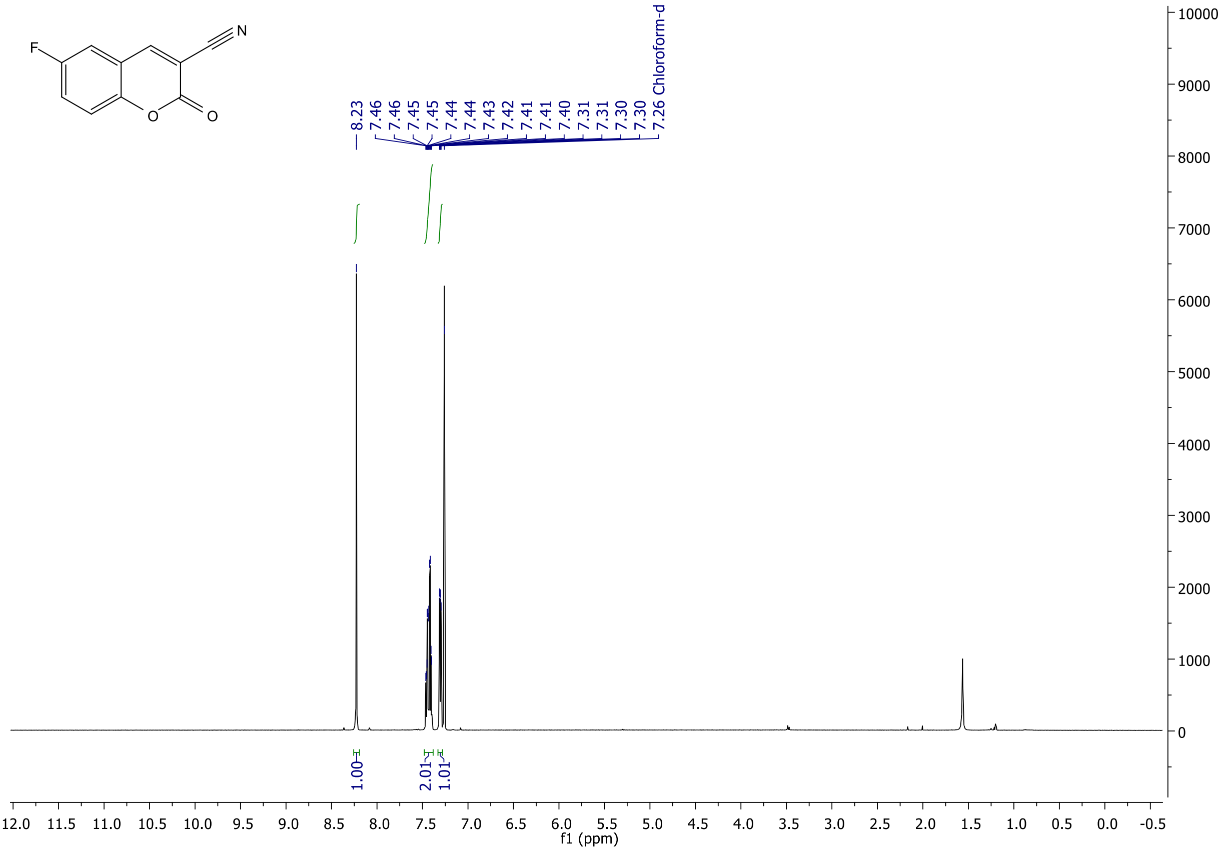

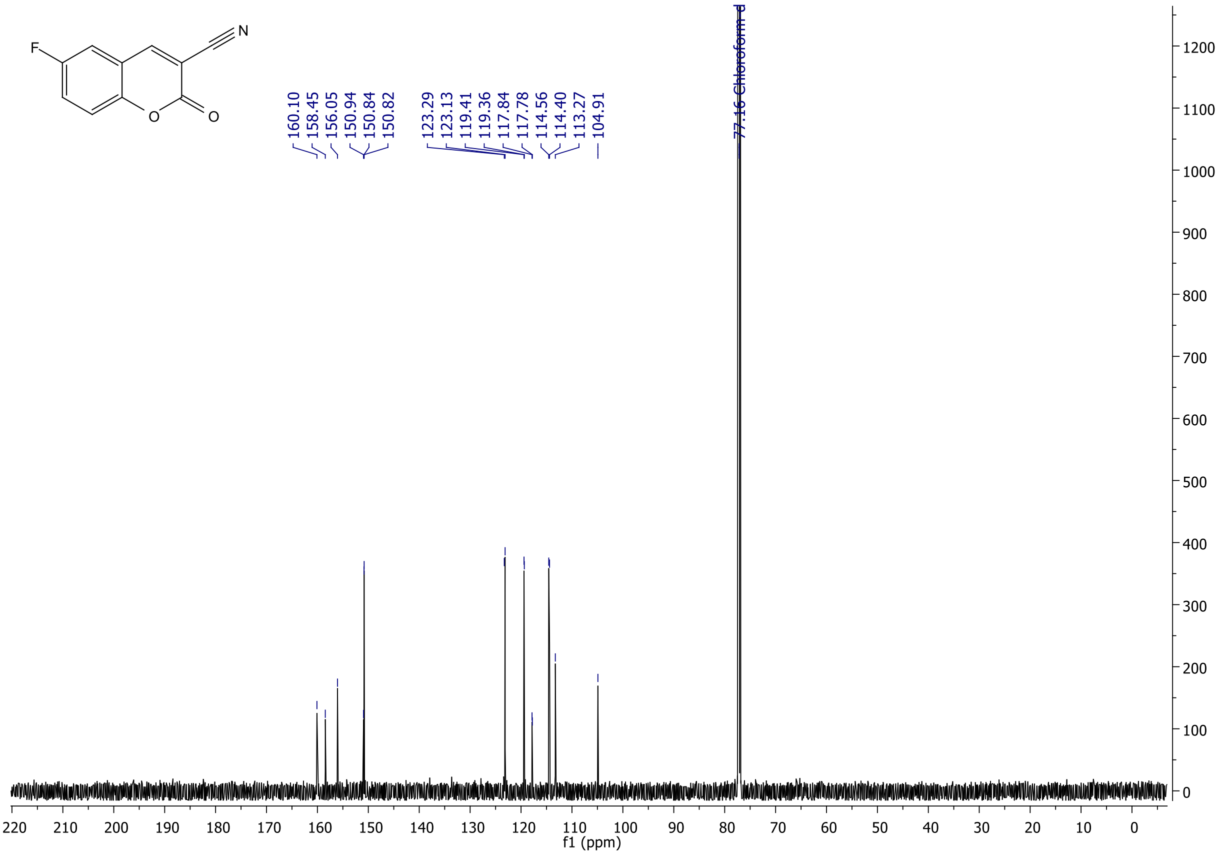

2b: Ethyl 6-chloro-2-oxo-2*H*-chromene-3-carboxylate

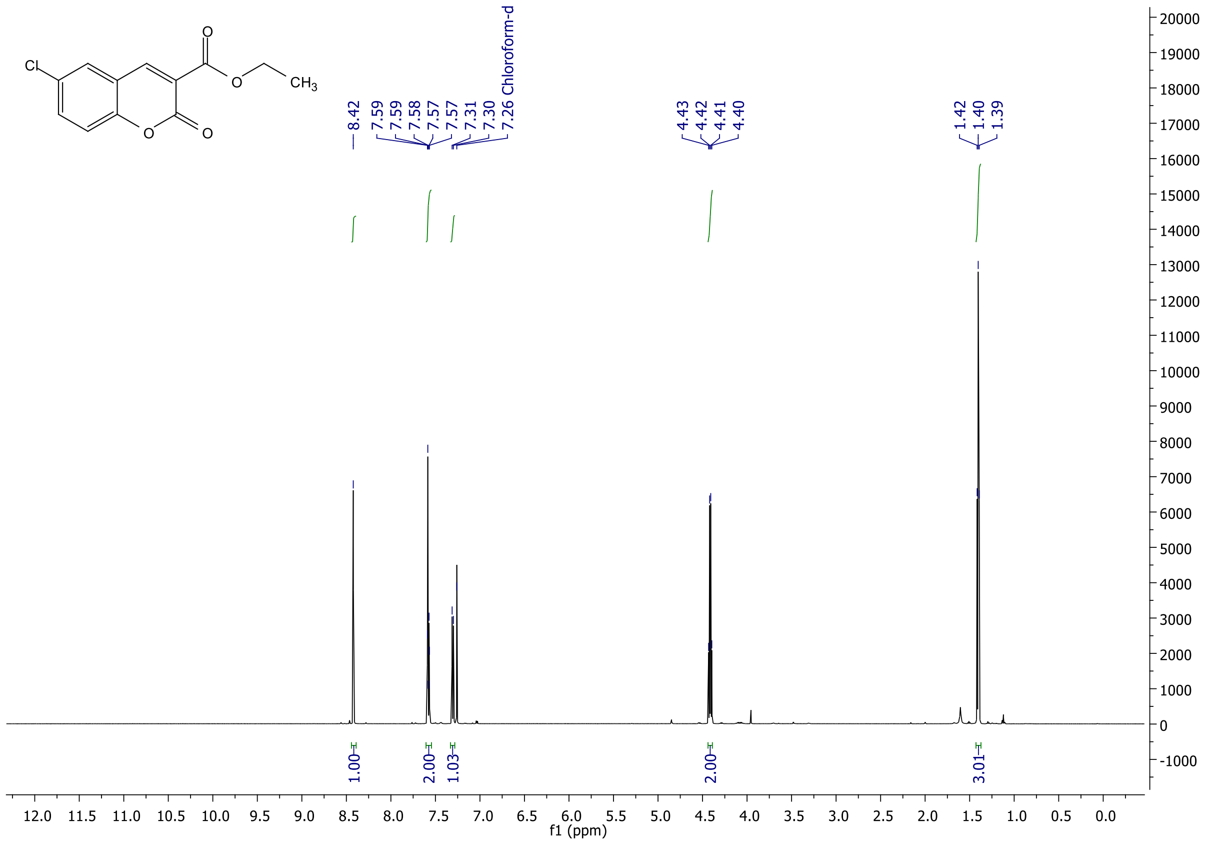

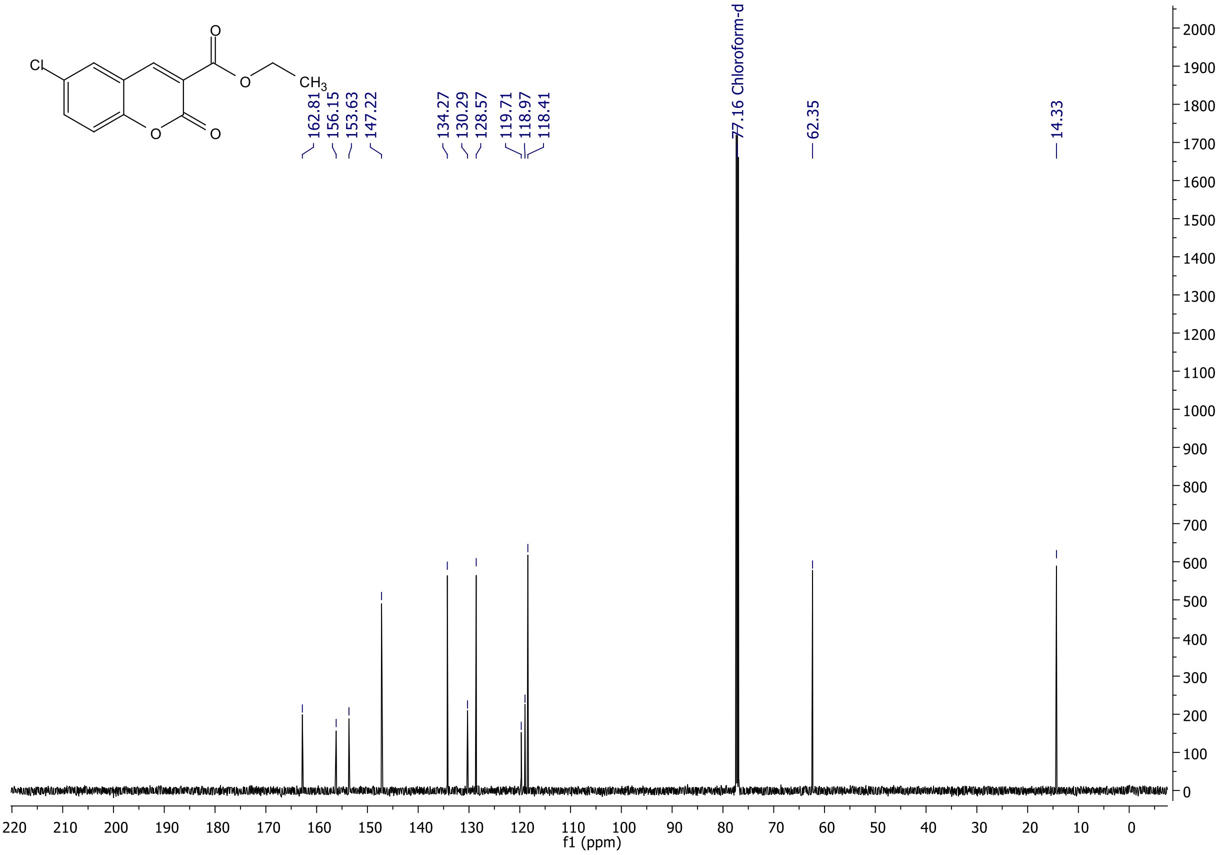

2c: 6-chloro-2-oxo-2*H*-chromene-3-carbonitrile

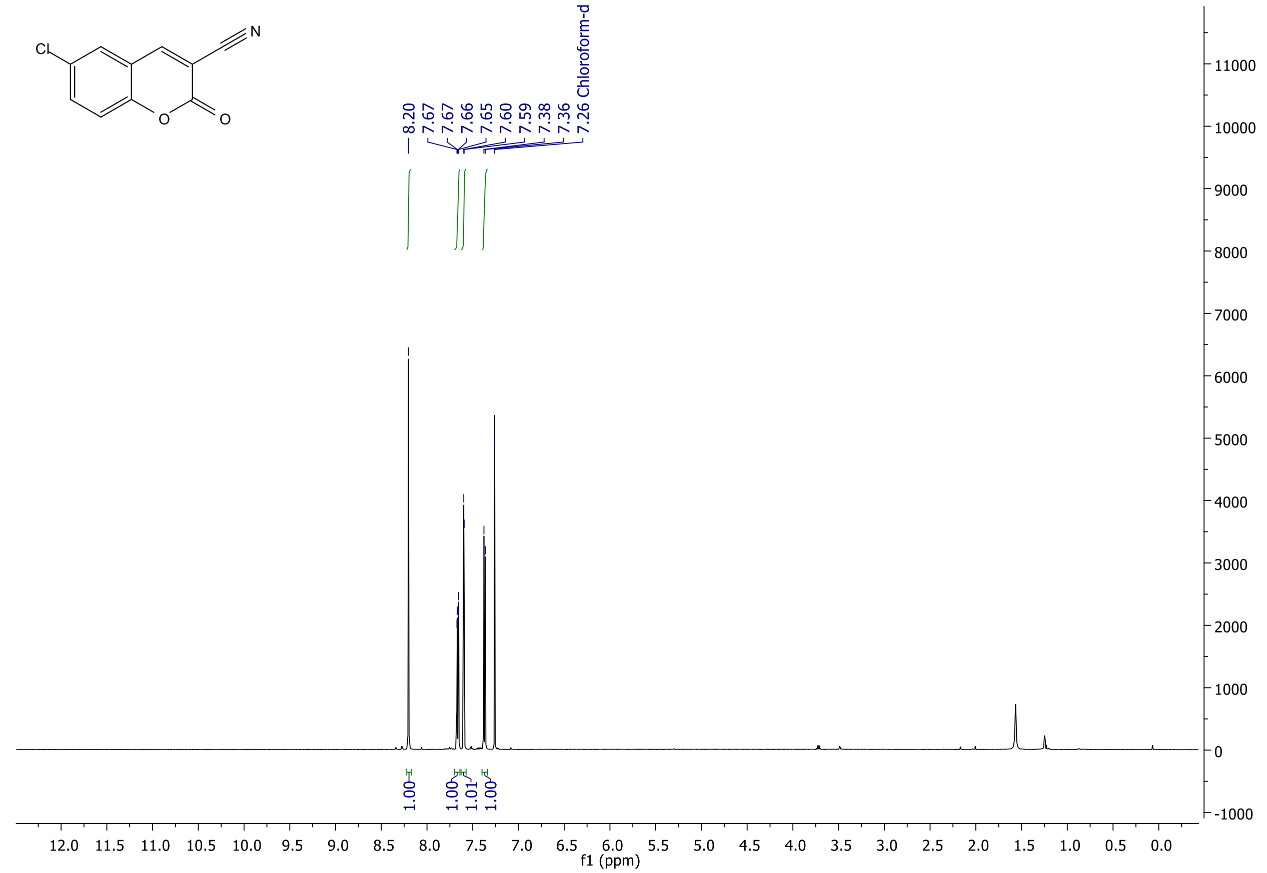

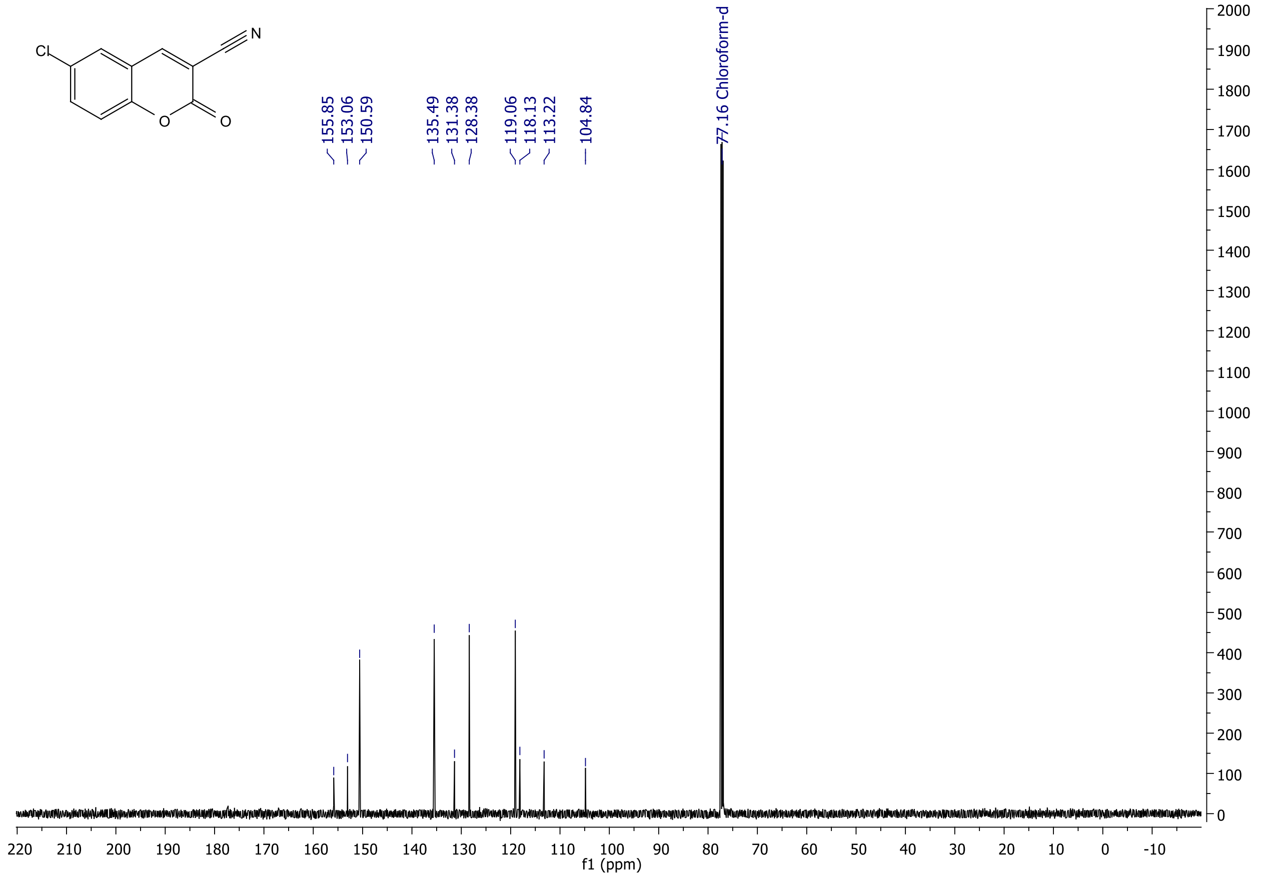

2d: Ethyl 6-bromo-2-oxo-2*H*-chromene-3-carboxylate

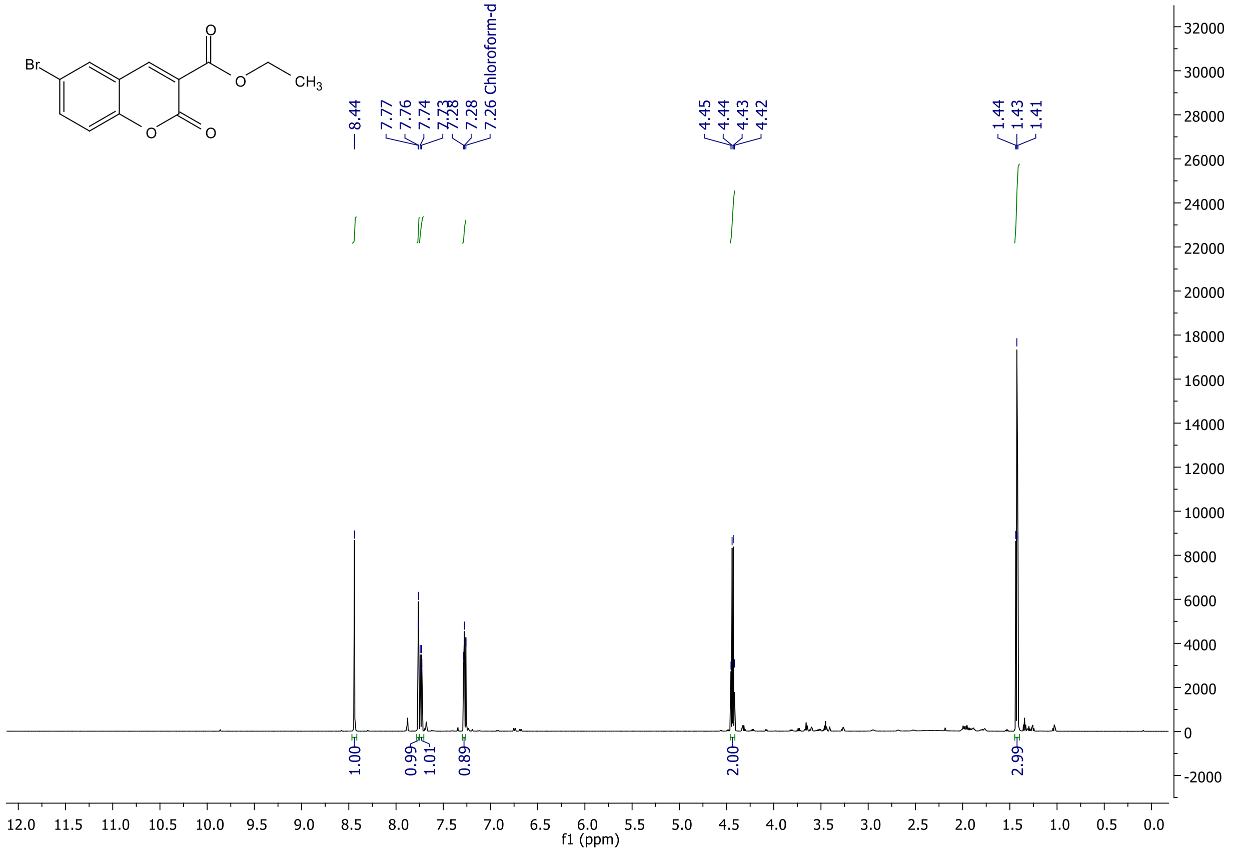

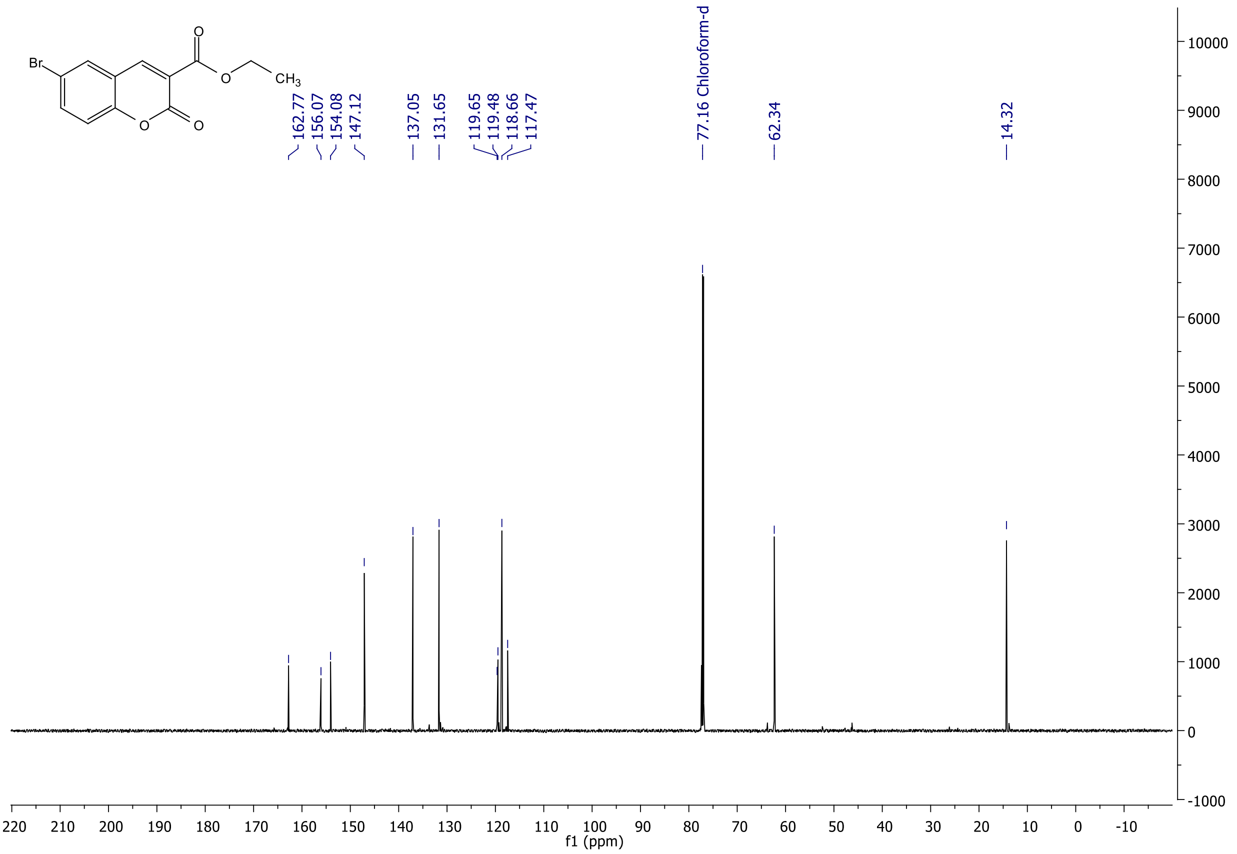

2e: 6-bromo-2-oxo-2*H*-chromene-3-carbonitrile

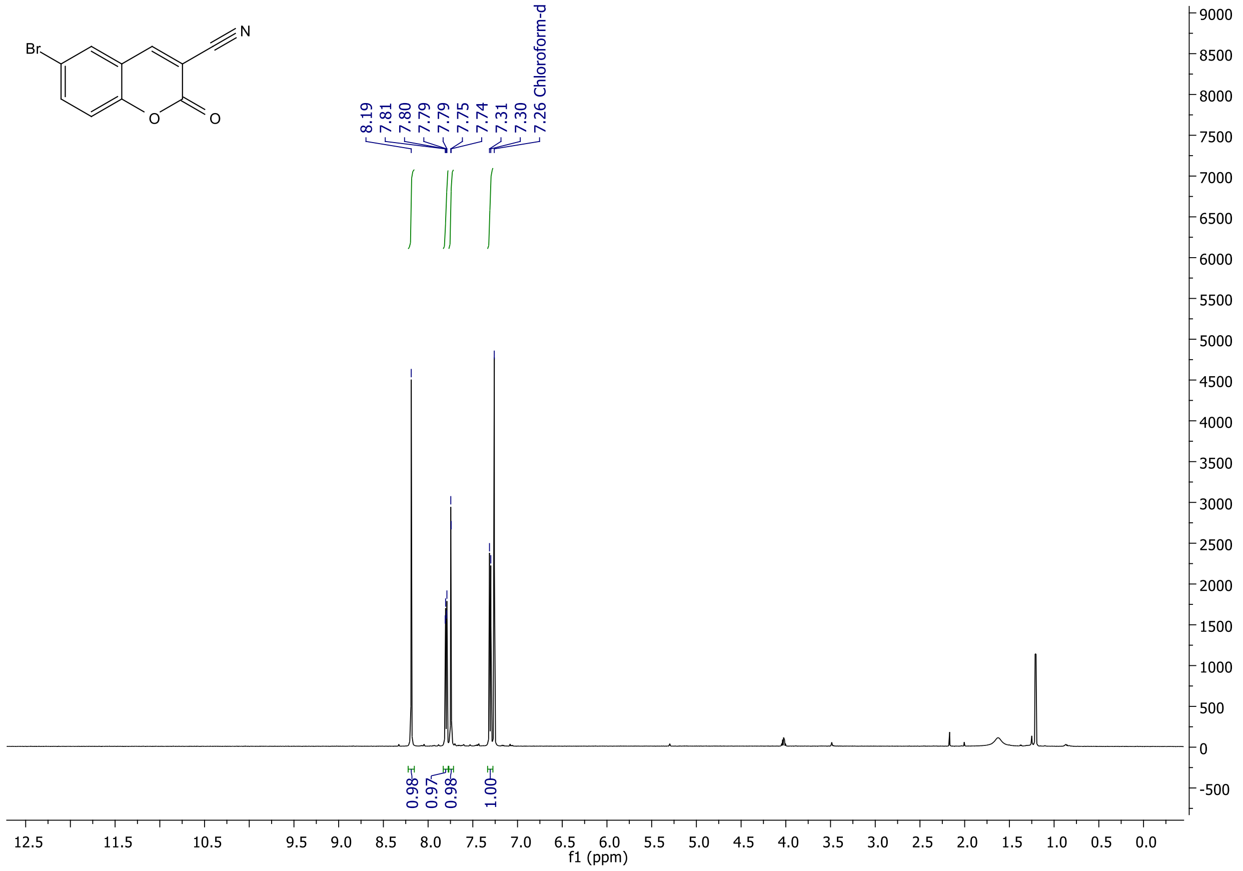

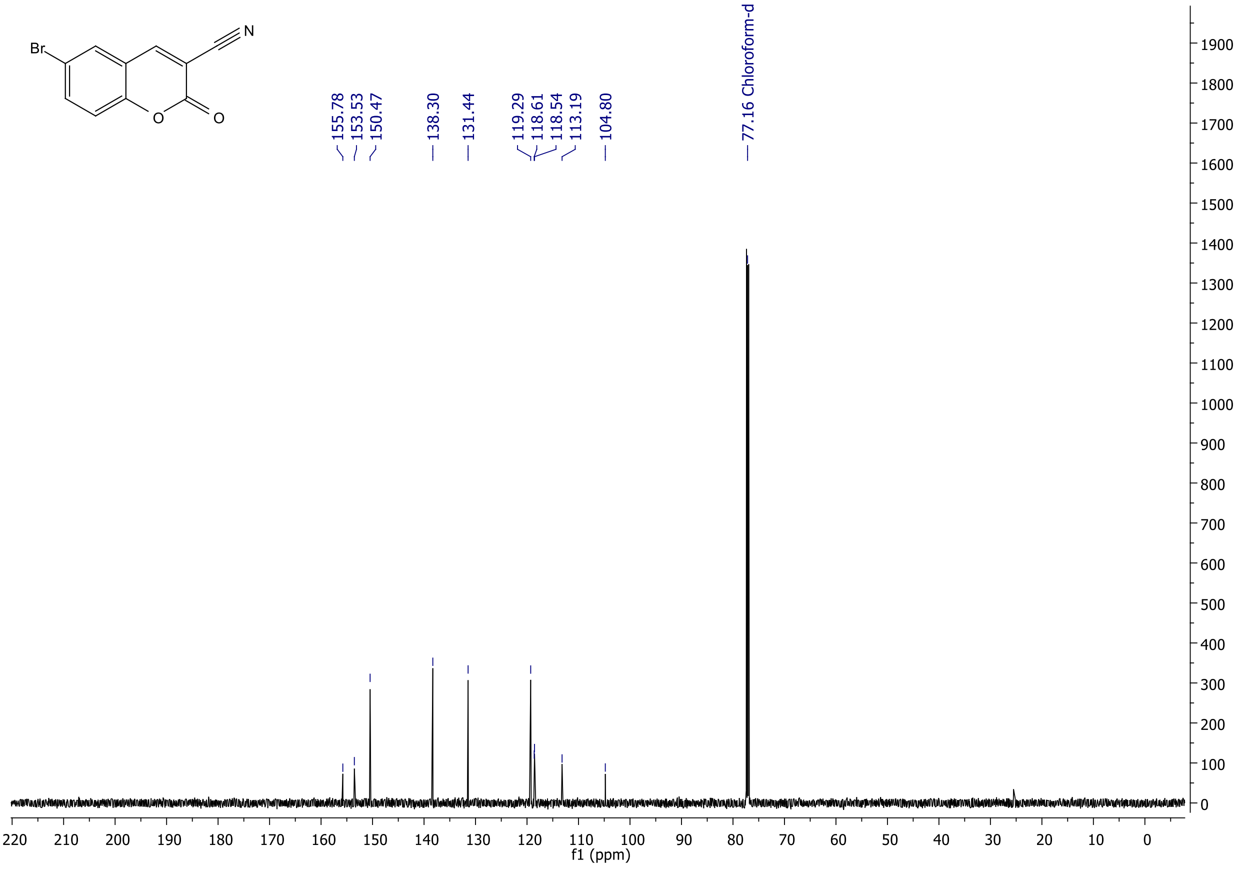

2f: 6-bromo-3-(methylsulfonyl)-2*H*-chromen-2-one

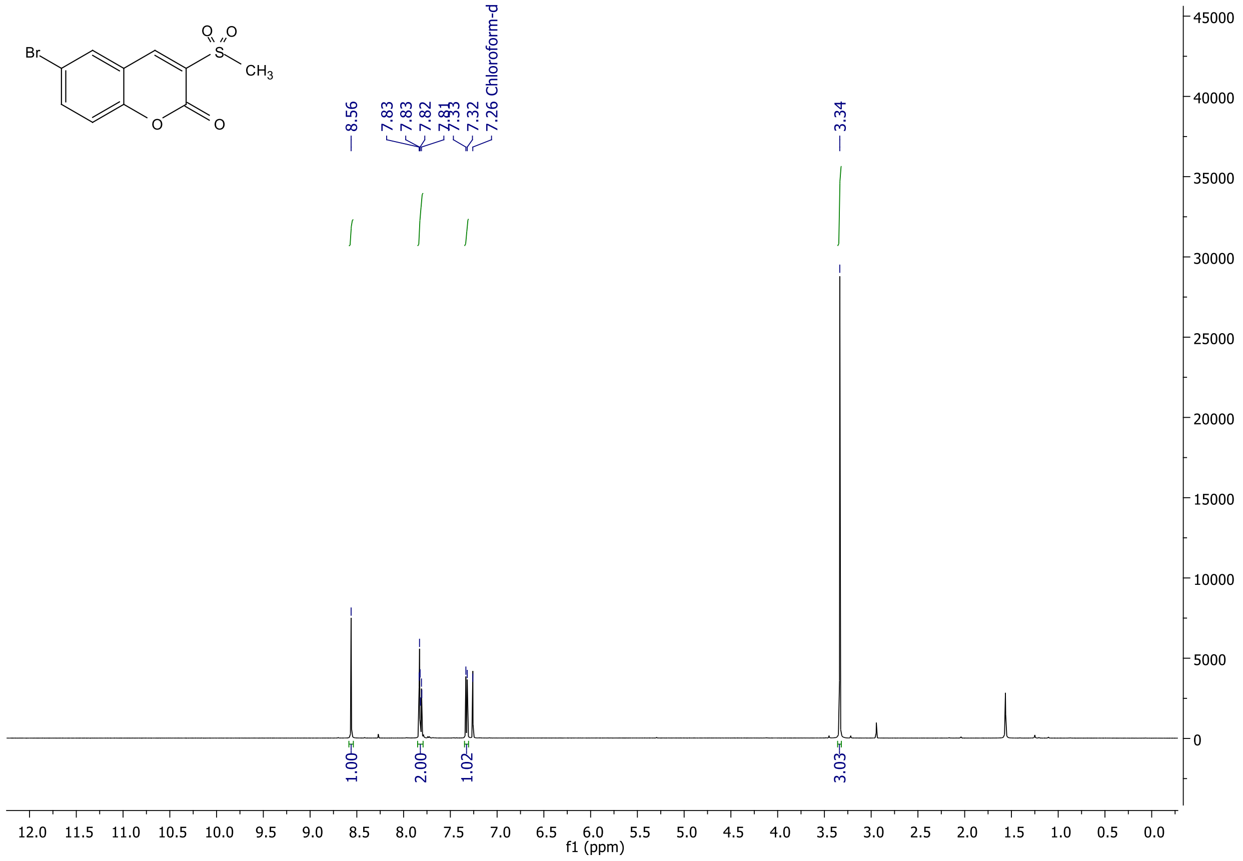

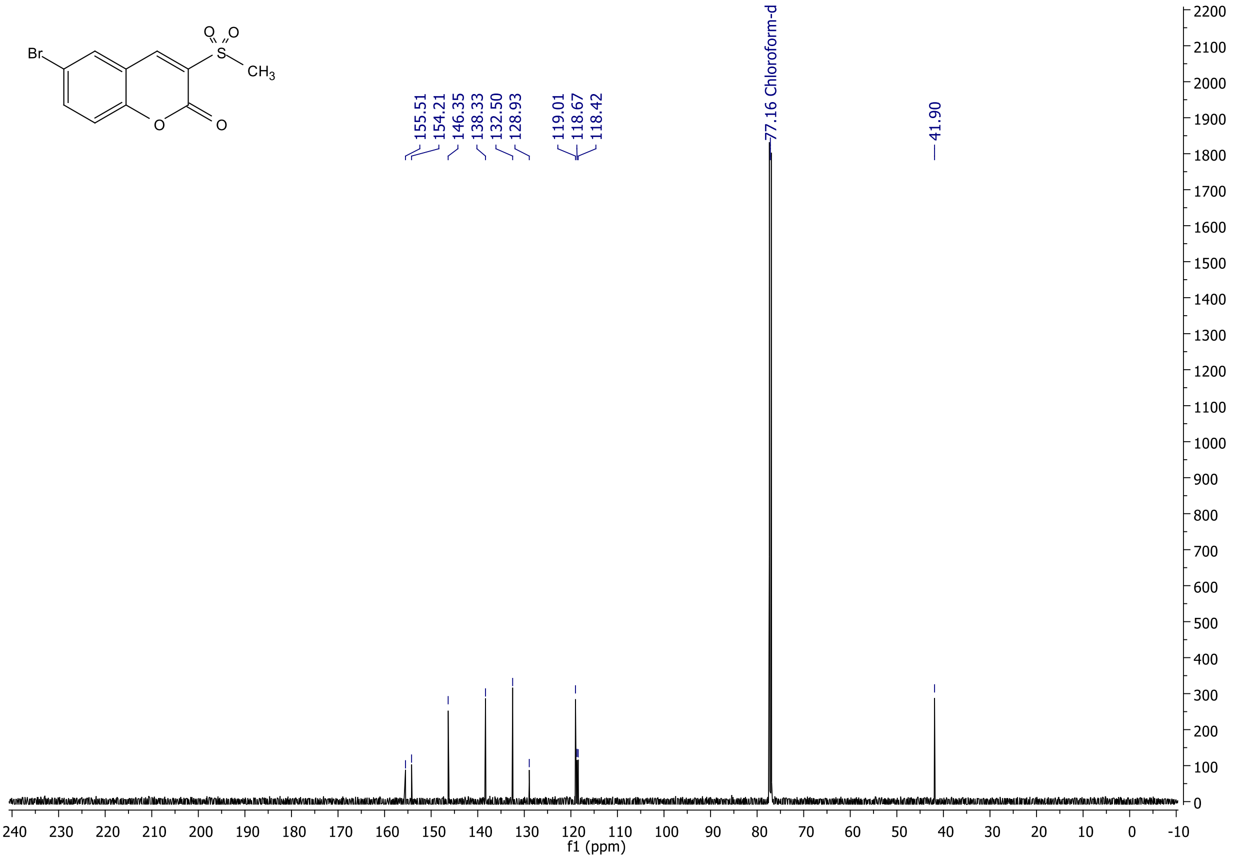

2g: Ethyl 6,8-dibromo-2-oxo-2*H*-chromene-3-carboxylate

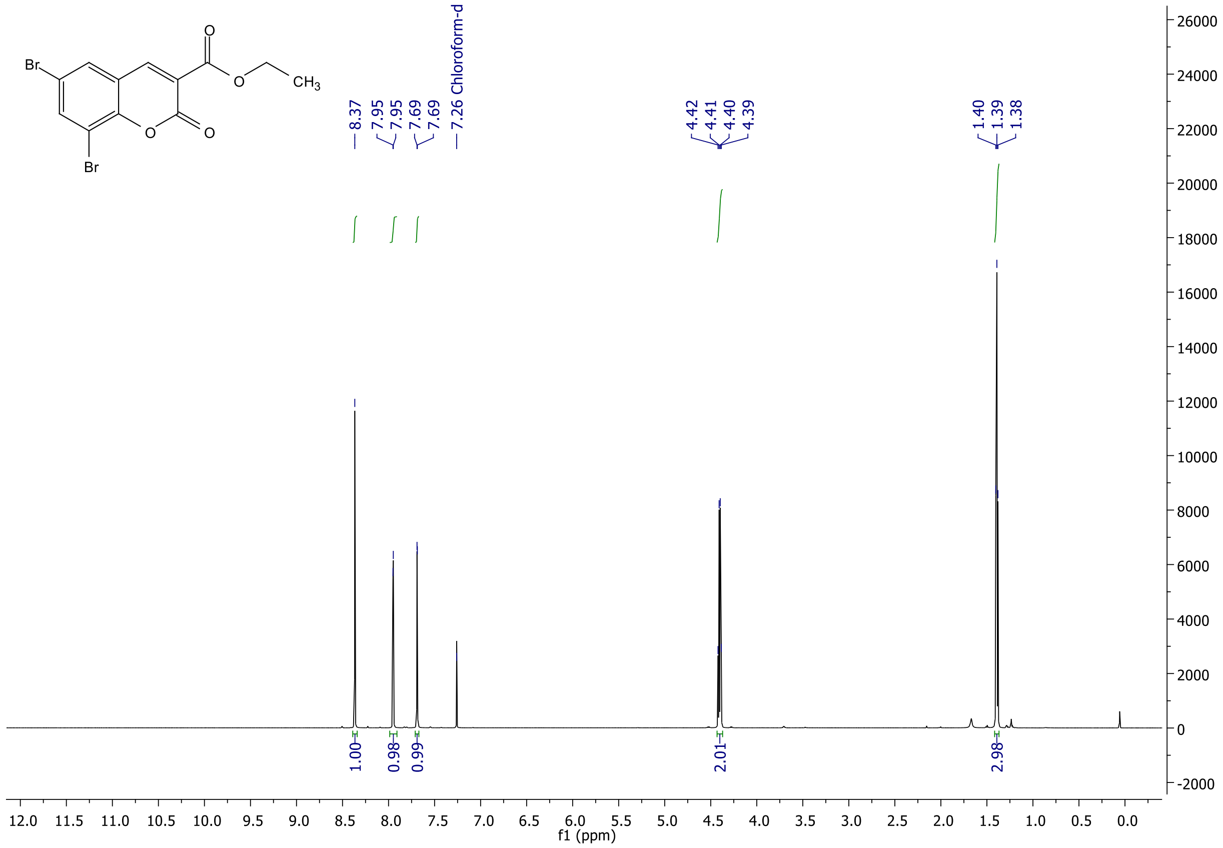

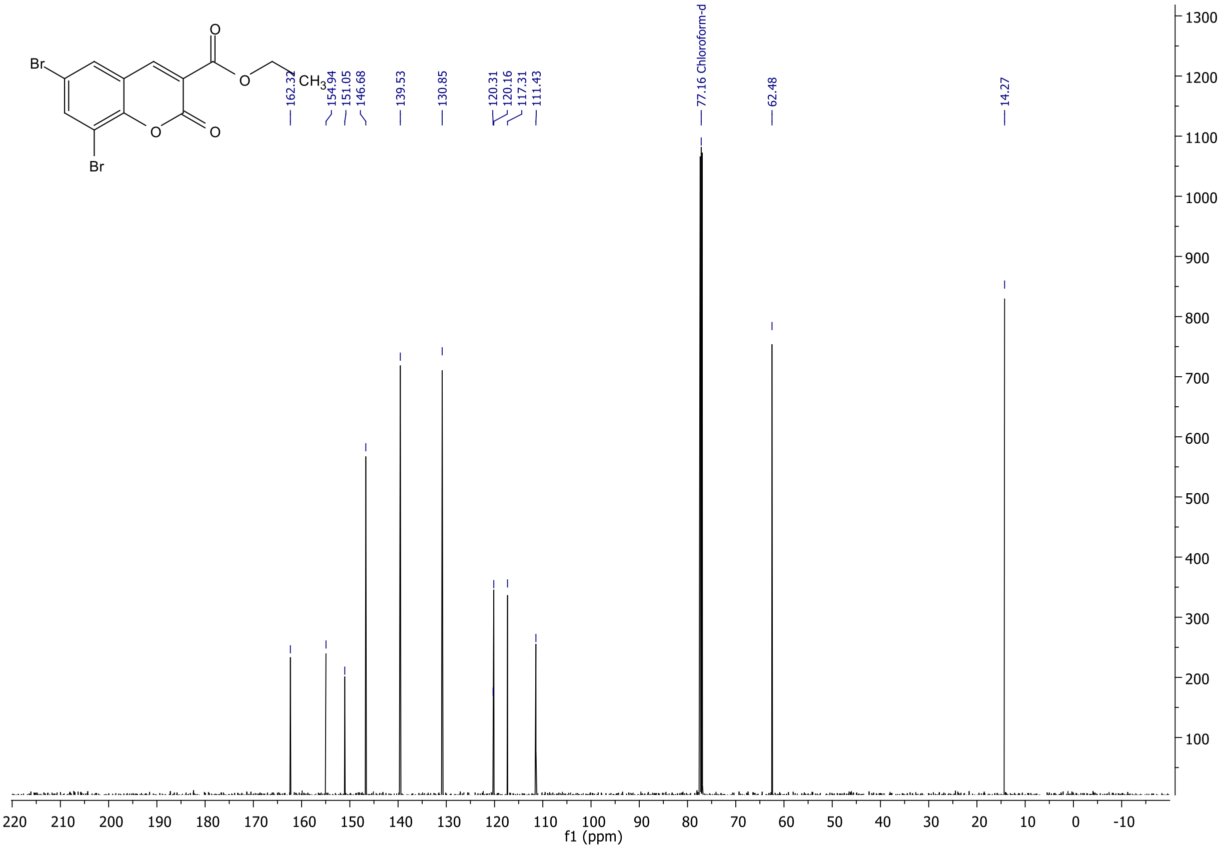

2h: 6,8-dibromo-2-oxo-2*H*-chromene-3-carbonitrile

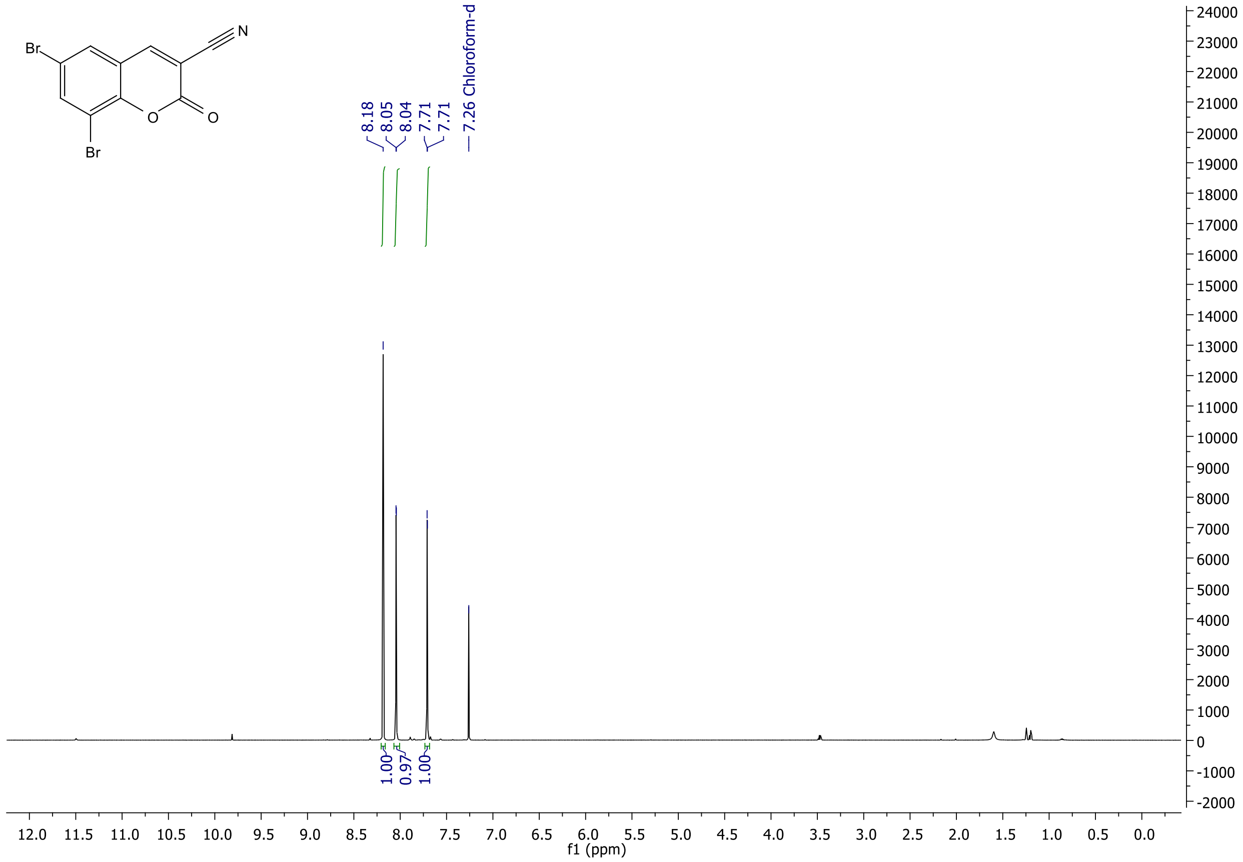

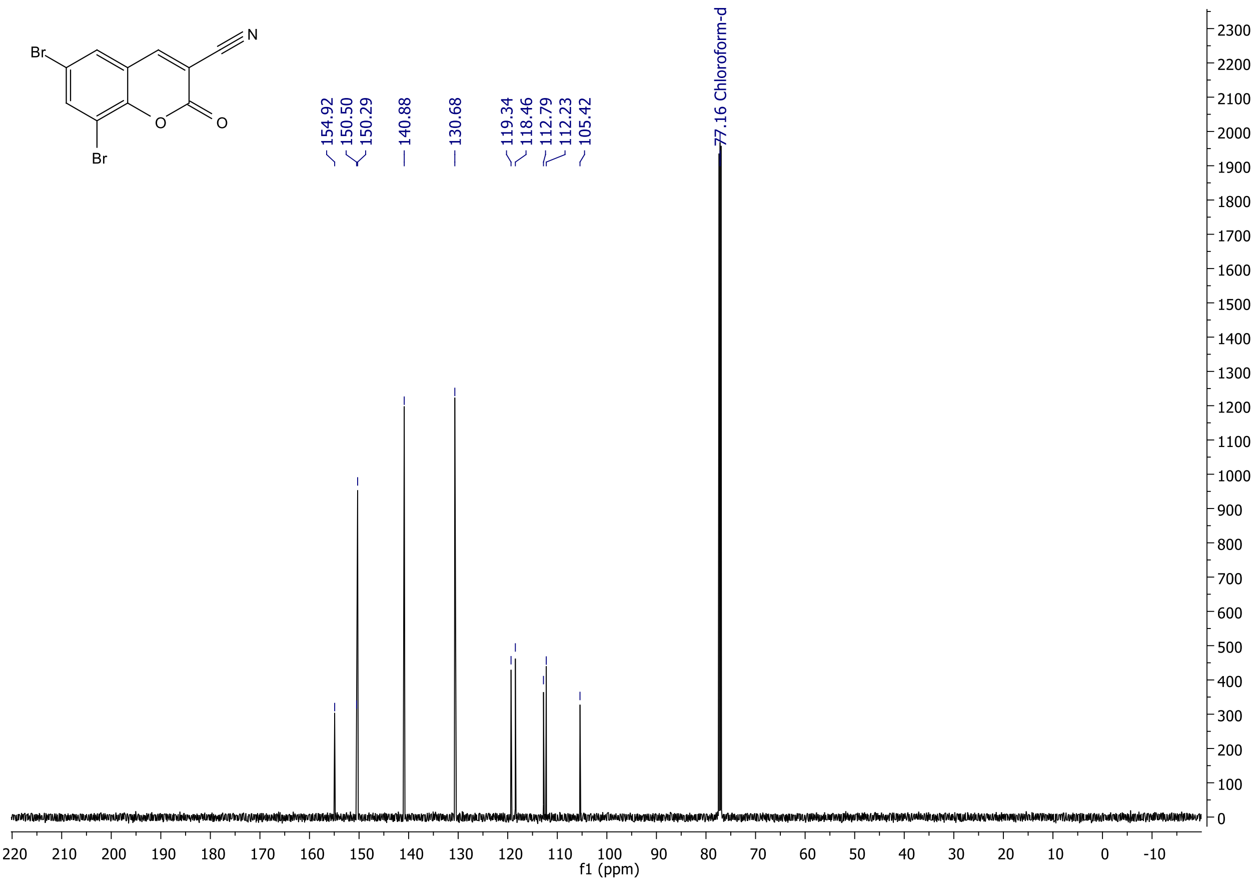

2i: ethyl 6,8-diiodo-2-oxo-2*H*-chromene-3-carboxylate

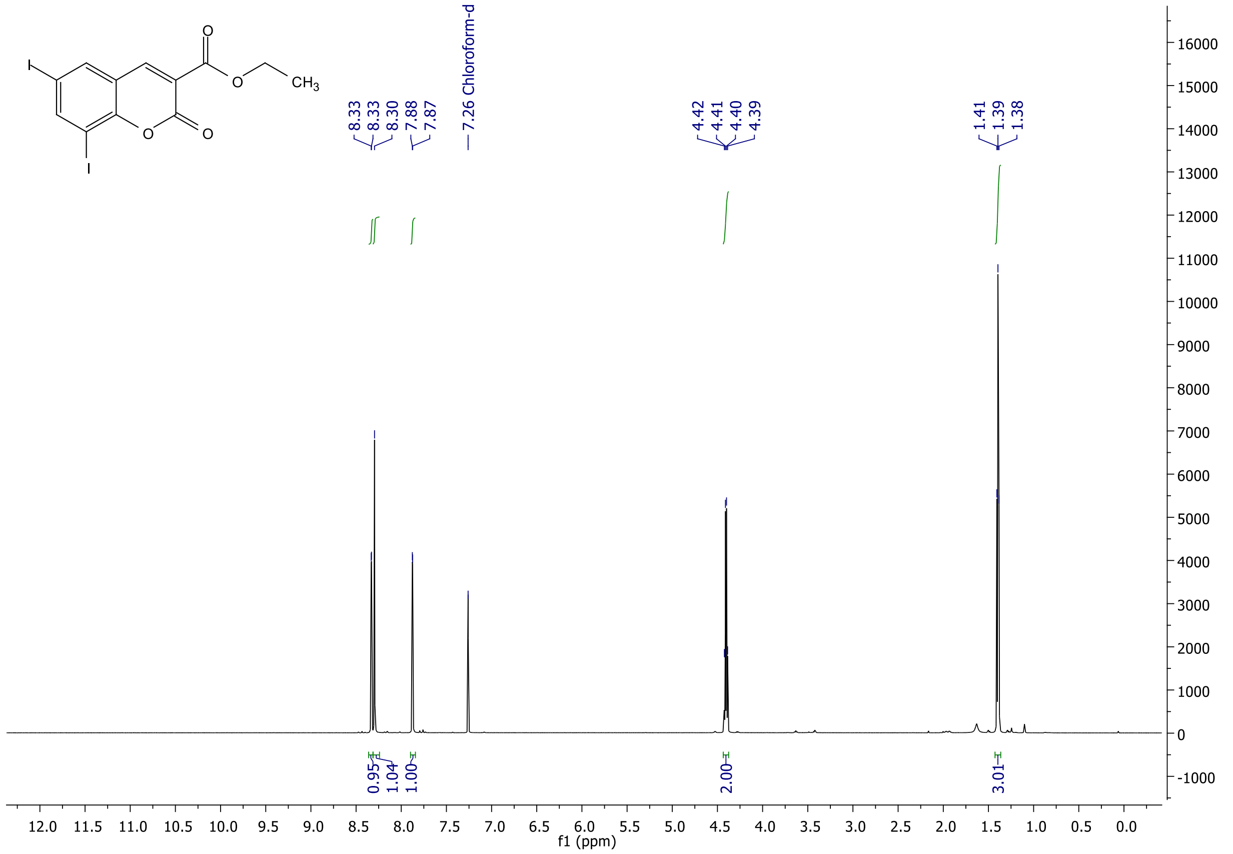

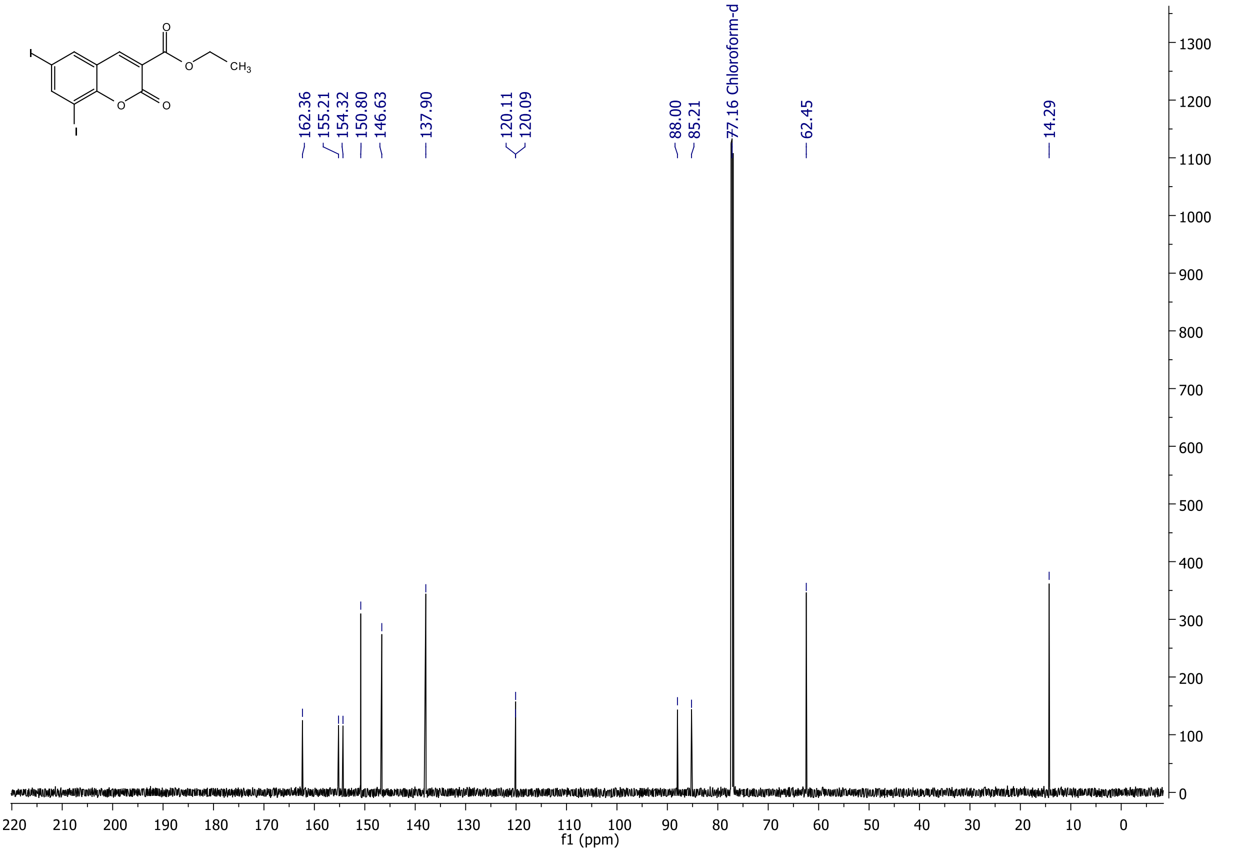

2j: 6,8-diiodo-2-oxo-2*H*-chromene-3-carbonitrile

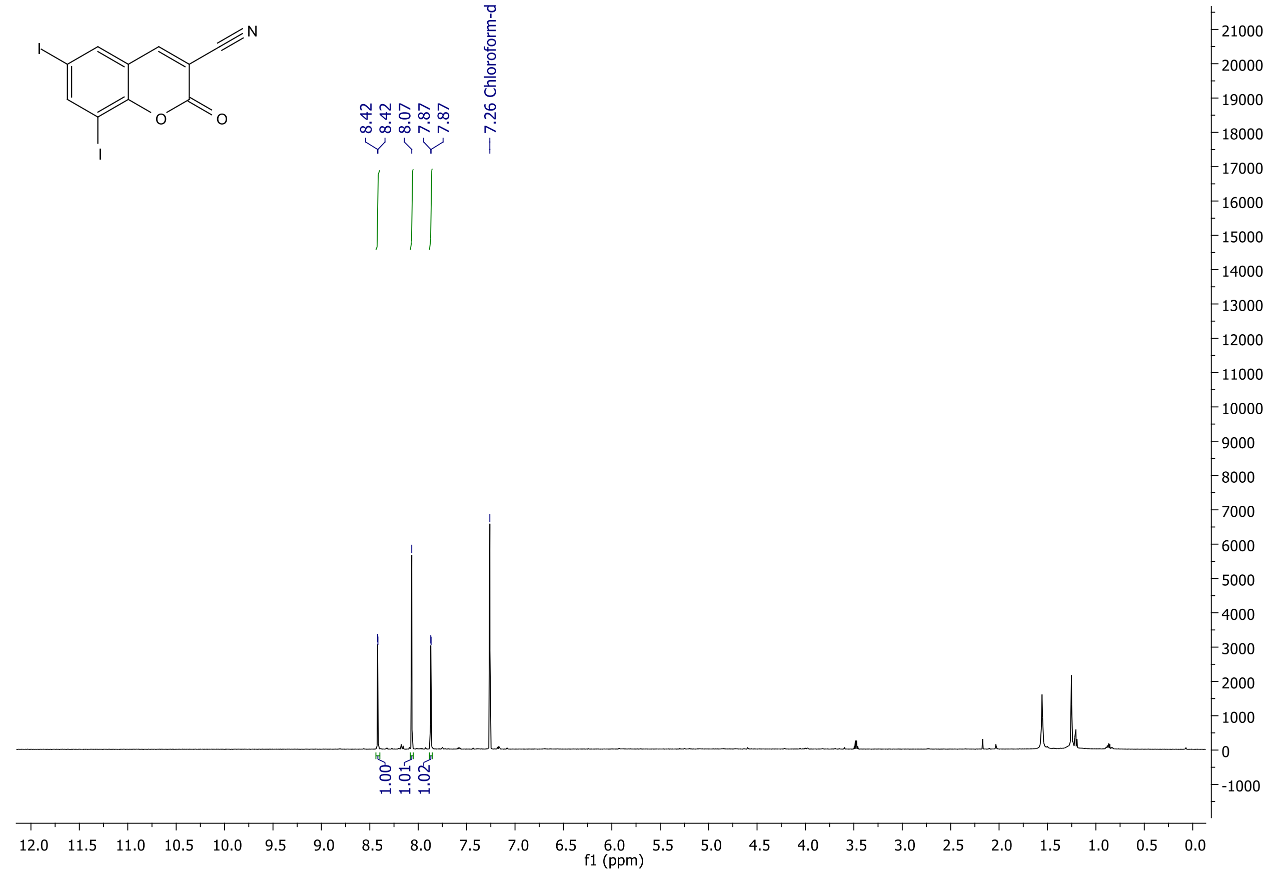

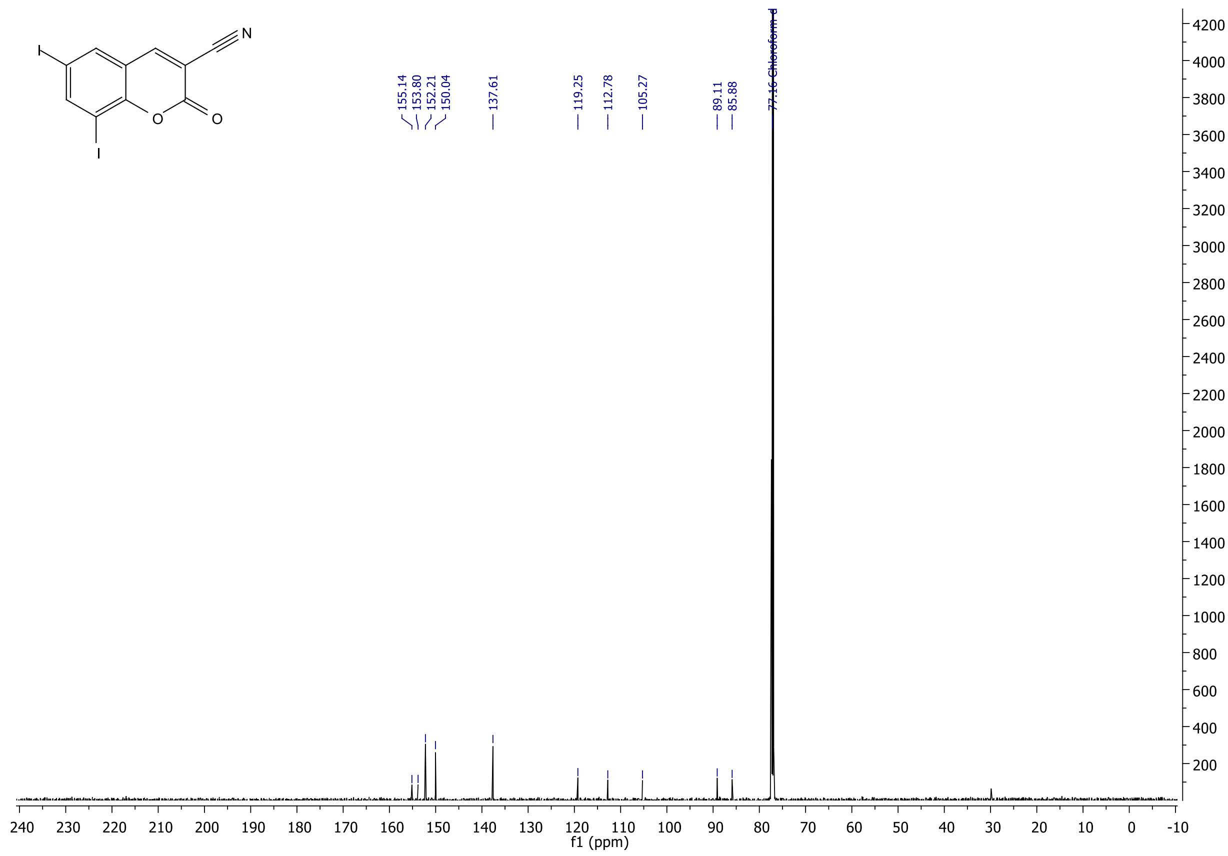

2k: 6,8-diiodo-2-oxo-2*H*-chromene-3-carboxylic acid

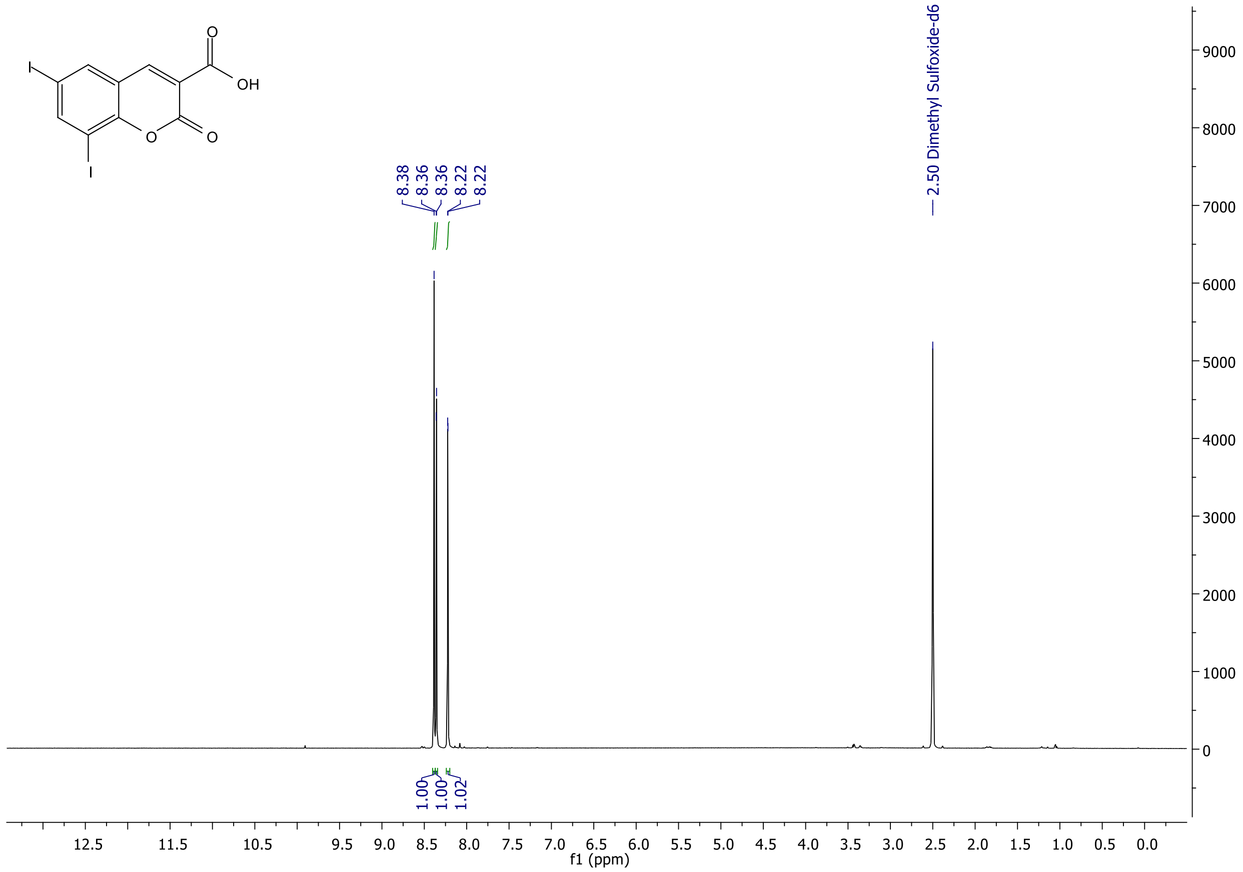

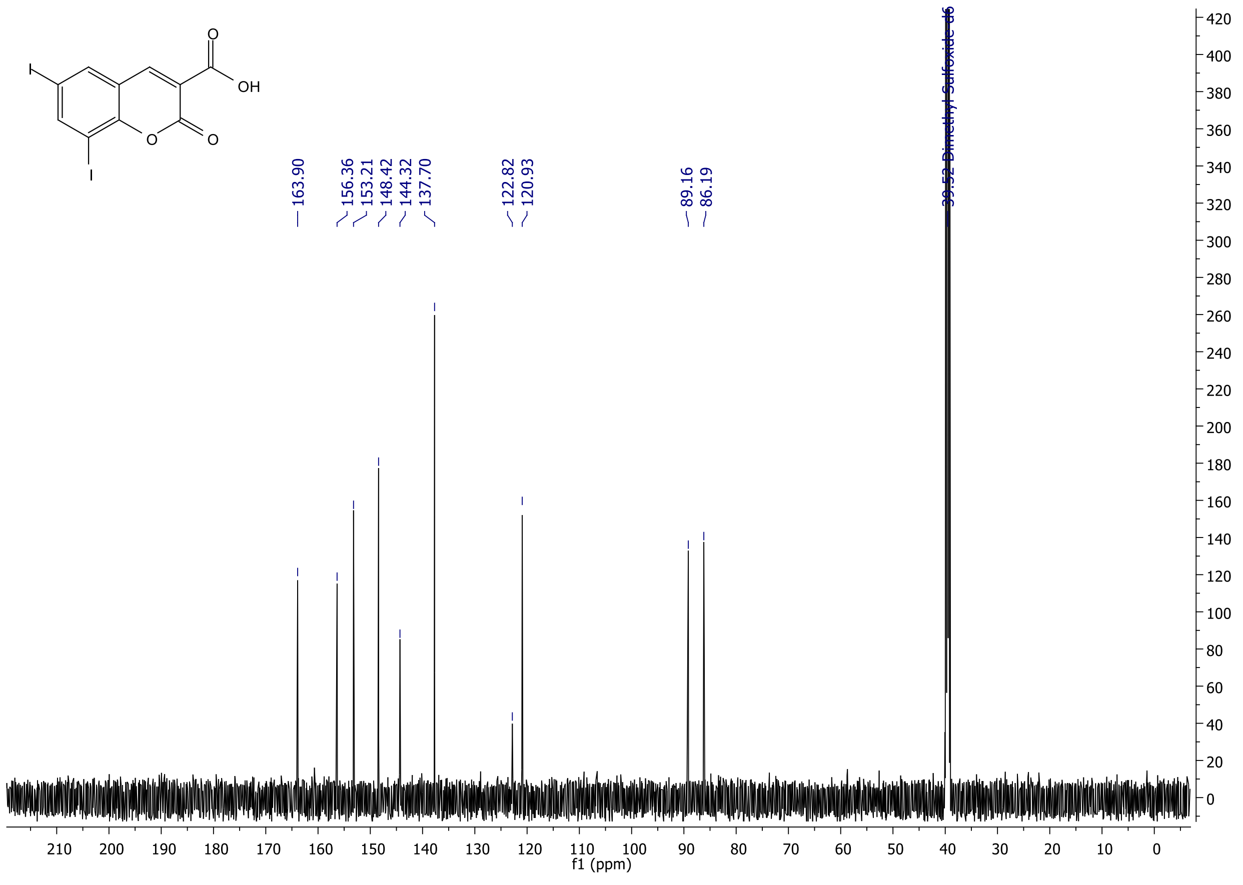

Supplement: Supplementary file 1 [file molecules-27-08897-s001.zip › molecules-2074296-supplementary.pdf]
